# Persistence of davemaoite at lower-mantle conditions

**DOI:** 10.1038/s41561-025-01657-9

**Published:** 2025-02-28

**Authors:** Lin Wang, Nobuyoshi Miyajima, Fei Wang, Tomoo Katsura

**Affiliations:** Bayerisches Geoinstitut, University of Bayreuth, Bayreuth, Germany

**Keywords:** Mineralogy, Petrology, Geochemistry

## Abstract

The lower mantle occupies over half of Earth’s volume, and accordingly, its mineralogy is crucial in determining the structure and dynamics of Earth. Davemaoite, the calcium silicate perovskite, was believed to coexist with bridgmanite in the lower mantle and is considered essential for understanding the chemical evolution and dynamics of Earth’s lower mantle. However, the presence of davemaoite is challenged due to the potential for high calcium silicate solubility in bridgmanite. Here we use an ultrahigh-pressure multi-anvil technique to show experimentally that the calcium solubility in bridgmanite is insufficient to eliminate davemaoite under mantle conditions, including typical mantle pressure, temperature and chemical compositions. We conclude that davemaoite has been stable in Earth’s lower mantle since its formation. Due to the limited calcium solubility in bridgmanite, davemaoite-enriched domains are expected at the core–mantle boundary. These domains could serve as the principal reservoir for incompatible elements in the lower mantle and may be the source for some ocean island basalts. Furthermore, our study offers an explanation for the observed large low-shear-wave-velocity provinces at the bottom of the lower mantle. These provinces may consist of davemaoite-enriched materials crystallized from basal magma ocean in early Earth history.

## Abstract (web summary)

The calcium silicate perovskite mineral davemaoite probably persisted since lower-mantle formation and may form davemaoite-enriched domains at the core–mantle boundary, according to an experimental study at lower-mantle conditions.

## Main

Davemaoite (Dvm), the calcium silicate (CaSiO_3_) perovskite, exhibits distinctive physical and chemical properties that contribute to our understanding of the chemical evolution and dynamics of Earth’s mantle. Its low seismic velocity offers a possible explanation for the observed low-seismic-velocity signatures^1^ and the large low-shear-wave-velocity provinces (LLSVPs)^2^ at the top and bottom of the lower mantle, respectively. Its low viscosity provides an intrinsic mechanism for delaminating slab materials and for accumulating recycled oceanic crust either at the boundary between the upper and lower mantles or at the core–mantle boundary^3^. Dvm is also well known as a geochemical reservoir for large-ion incompatible elements in the lower mantle^4^ and inferred to contribute to the geochemical diversity observed in ocean island basalts^5,6^. It is the last crystallized phase during magma ocean solidification^7–9^ and therefore affects the element distribution in Earth’s mantle.

However, the fundamental question of the existence of Dvm in the lower mantle remains under debate. Some studies have suggested that the solubility of CaSiO_3_ in (Mg,Fe)(Al,Si)O_3_ bridgmanite (Bdm, the most abundant mineral in the lower mantle), expressed as *χ*_Ca_, here defined as the Ca content in cation units normalized to 2, is too low to make Dvm completely dissolve into Bdm^10–12^. These studies proposed that Dvm is the third most abundant phase in the lower mantle^10,13,14^. Other studies^15–17^, however, have reported that *χ*_Ca_ increases markedly with temperature or Fe content in Bdm, leading to the conclusion that Dvm is absent in hot or oxidized regions in Earth’s lower mantle. Therefore, it remains uncertain whether Dvm exists in the lower mantle.

In this Article, we systematically investigate the *χ*_Ca_ under the lower-mantle conditions using our ultrahigh-pressure multi-anvil technique^18^ at pressures from 27 to 50 GPa and temperatures from 2,300 to 2,700 K. Five different starting materials (Extended Data Table 1) with compositions of Ca_0.5_Mg_0.5_SiO_3_ (Ca50), Ca_0.04_Mg_0.85_Fe_0.10_SiO_3_ (Ca4Fe10), Ca_0.08_Mg_0.80_Fe_0.10_SiO_3_ (Ca8Fe10), Ca_0.08_Mg_0.70_Fe_0.11_Al_0.11_Si_0.98_O_3.1_ (Fe11Al11) and Ca_0.5_Mg_0.3_Fe_0.2_Al_0.2_Si_0.8_O_3_ (Fe20Al20) are used to study the composition effect. Details of our experimental procedures can be found in Methods. Our results demonstrate that *χ*_Ca_ of Bdm is very low, less than 0.03 per 2-cation formula unit (p.f.u.), and insensitive to other compositional parameters. Therefore, Dvm should be present throughout the entire lower mantle.

## Coexistence of bridgmanite and davemaoite

**Fig. 1:**
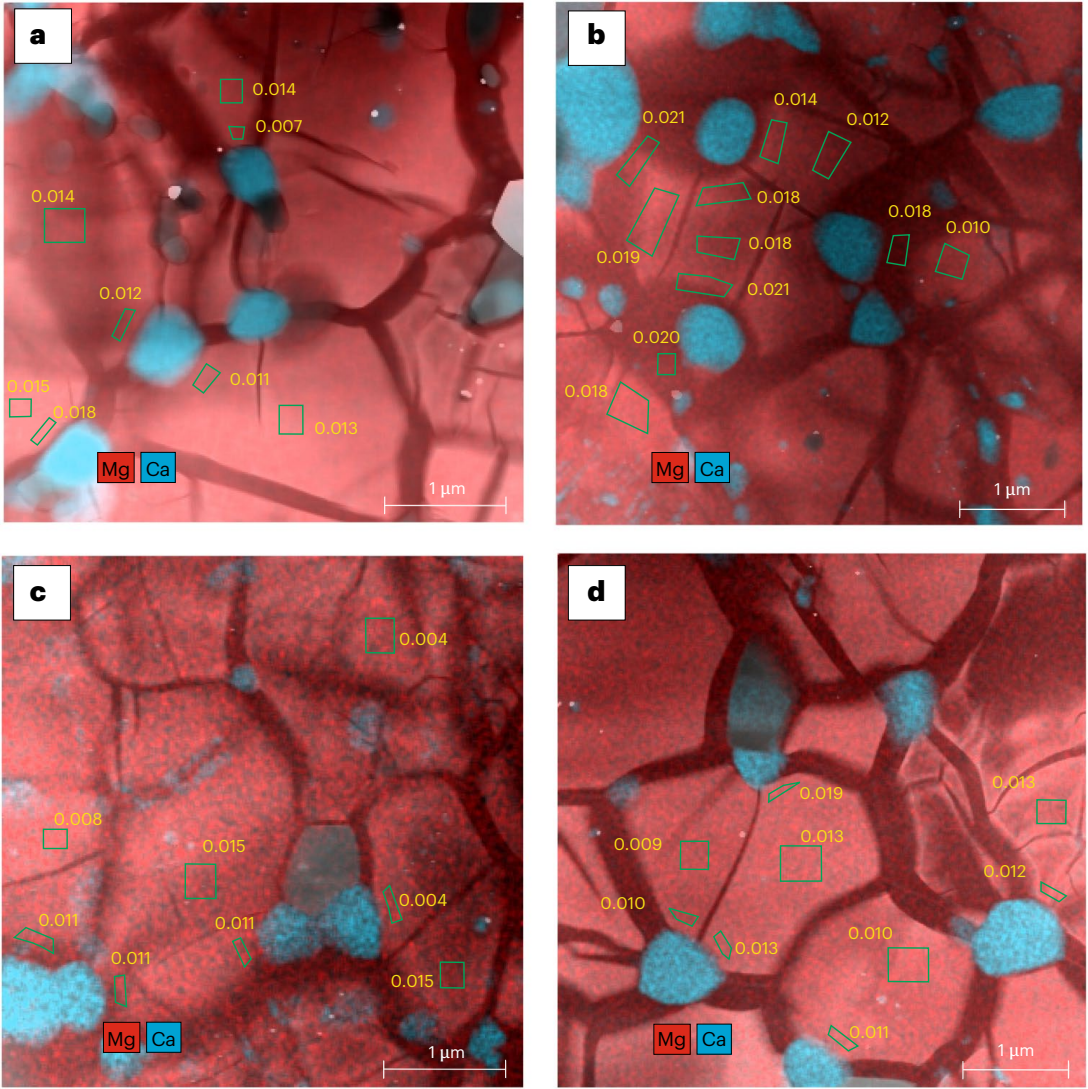
Compositional maps of Ca4Fe10 and Ca8Fe10 samples formed under 2,600–2,700 K and 40–50 GPa. **a**, Ca4Fe10, 40 GPa, 2,600 K. **b**, Ca8Fe10, 40 GPa, 2,700 K. **c**, Ca8Fe10, 50 GPa, 2,600 K. **d**, Ca8Fe10, 50 GPa, 2,700 K. High-angle angular dark-field STEM images are superimposed with EDS chemical maps. The blue and red colours, respectively, indicate concentrations of Ca and Mg, which should correspond to the distributions of Dvm and Bdm grains, respectively. The yellow numbers on the map are the Ca p.f.u. in Bdm in the nearby green selected area. The core and rim parts in a single grain show similar Ca contents for Bdm.

**Extended Data Fig. 1:**
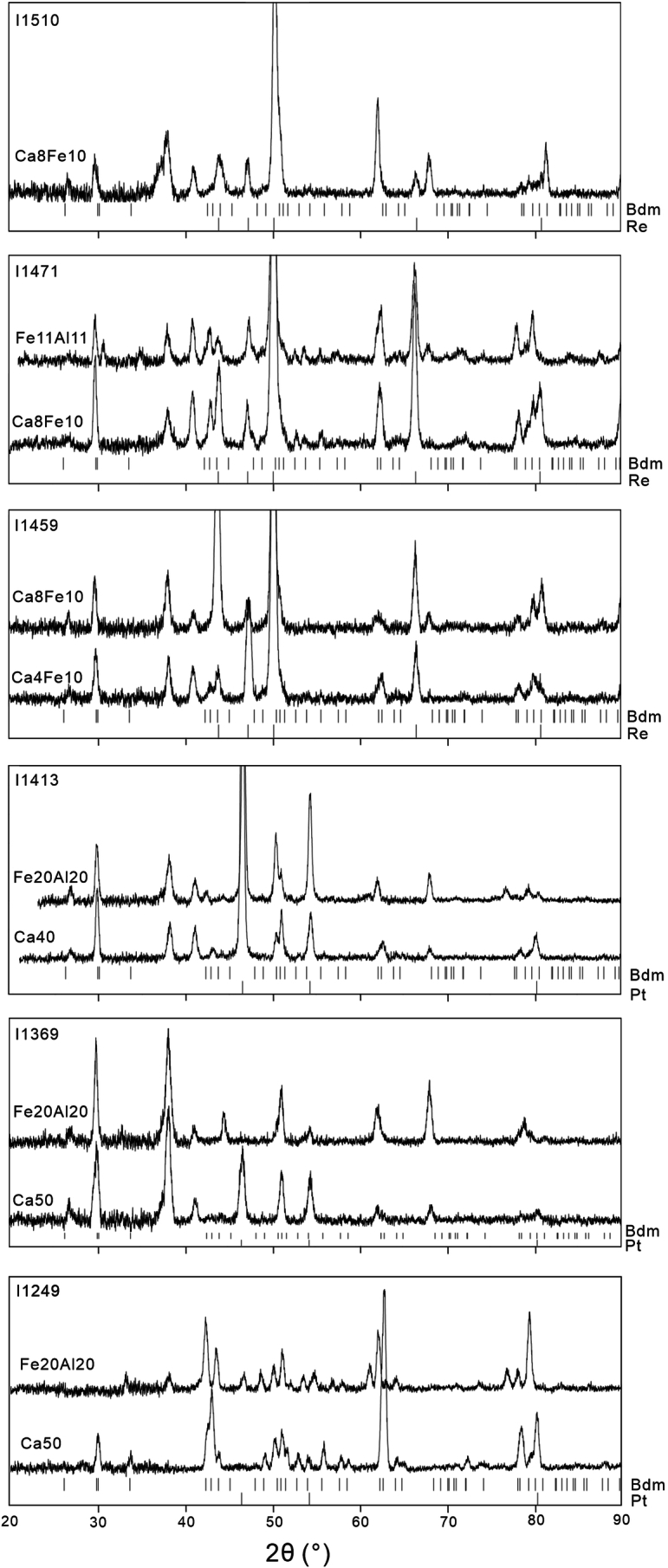
XRD pattern of run products in this study. The positions of diffraction peaks from bridgmanite, Re and Pt are listed at the bottom of each panel; 2θ is the angle between the incident and diffracted X-ray beams.

**Extended Data Fig. 2:**
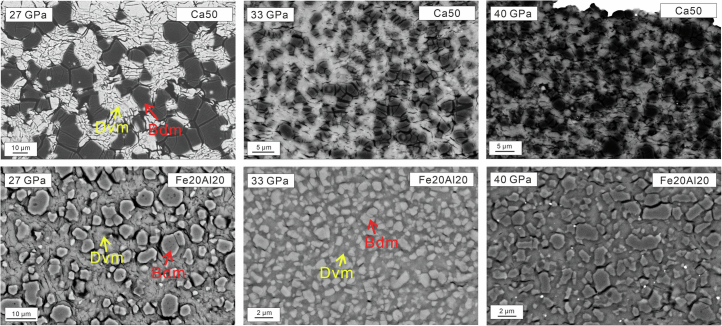
BSE images of Ca50 and Fe20Al20 sample synthesized at 2300 K, 27-40 GPa. Bridgmanite and davemaoite are clearly distinguished by the color contrast.

**Extended Data Fig. 3:**
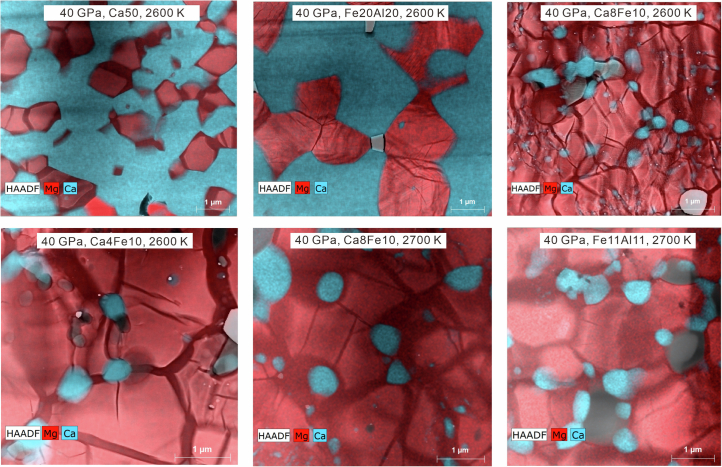
Compositional maps of samples synthesized under 2600-2700 K and 40 GPa. The experimental conditions and bulk composition are listed in each figure. High-angle angular dark-field (HAADF) scanning TEM images superimposed with EDS chemical maps. The blue and red colors indicate concentrations of Ca, and Mg, which should correspond to Dvm and Bdm.

Bdm was identified in all run products using X-ray diffraction (XRD) patterns (Extended Data Fig. 1). By contrast, Dvm was not visible by XRD due to amorphization upon decompression^12^. Bdm and Dvm were both identified in back-scattered electron images (Extended Data Fig. 2) and scanning transmission electron microscopy (STEM; Fig. 1 and Extended Data Fig. 3). For runs conducted at 2,300 K, Bdm in the Ca50 samples showed typical cleavage-like texture (Extended Data Fig. 2). Bdm has very sharp grain boundaries while Dvm forms an interconnected matrix without any clear grain boundaries. For runs conducted at temperatures of 2,600–2,700 K, the composition mapping from STEM–energy-dispersive X-ray spectroscopy (EDS) (Fig. 1 and Extended Data Fig. 3) clearly shows two distinct phases with very different but homogeneous Ca contents, indicating the coexistence of both Bdm and Dvm in these runs.

## Limited Ca solubility in bridgmanite

**Fig. 2:**
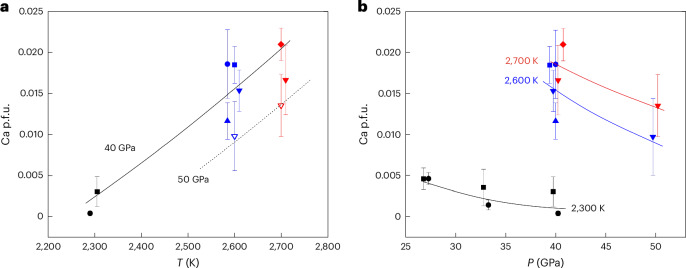
Temperature and pressure dependence of *χ*_Ca_ in Bdm. **a**, Temperature (*T*) dependence. **b**, Pressure (*P*) dependence. The circle, triangle, inverted triangle, diamond and square symbols represent Bdm from Ca50, Ca4Fe10, Ca8Fe10, Fe11Al11 and Fe20Al20 samples, respectively. The black, blue and red colours represent Bdm synthesized at 2,300, 2,600 and 2,700 K. The solid and open symbols in **a** represent 40 GPa and 50 GPa runs, respectively. The data points are offset slightly from their true *x* values to avoid overlapping of symbols and error bars. The lines are guides for the eye. The 2*σ* error bars are shown. The sample size (*n*) for each point is listed in Extended Data Table 2.

The *χ*_Ca_ of Bdm increases roughly and by less than 0.02 with temperature from 2,300 to 2,700 K (Fig. 2a). By contrast, the *χ*_Ca_ of Bdm decreases with increasing pressure (Fig. 2b). At 2,300 K, *χ*_Ca_ decreases by 0.002 and 0.005 p.f.u. from 27 GPa to 40 GPa in the Ca50 and Fe20Al20 samples, respectively. At 2,600 K and 2,700 K, *χ*_Ca_ of Bdm decreases by 0.005 from 40 to 50 GPa.

Fe and Al have a trivial effect on *χ*_Ca_ (Fig. 2). At a temperature of 2,300 K, the Fe in Bdm is predominantly Fe^3+^ (Extended Data Table 2), and it couples with Al to form the FeAlO_3_ component in Bdm^19^. The FeAlO_3_ component is found to increase *χ*_Ca_ only slightly (Fig. 2b). At 2,600 K and 2,700 K, Fe in Bdm is mainly Fe^2+^ (Extended Data Table 2). The *χ*_Ca_ of pure MgSiO_3_ Bdm is nearly the same as *χ*_Ca_ of Fe-bearing Bdm and Fe, Al-bearing Bdm within errors (Fig. 2), demonstrating the small effect of Fe and Al on the *χ*_Ca_ of Bdm at high temperatures.

It is essential to emphasize that *χ*_Ca_ is exceedingly low under the current experimental conditions. Even at our highest temperature (2,700 K), *χ*_Ca_ is less than 0.025 at 40 and 50 GPa. This is considerably less than 0.08, which is the value required for the CaSiO_3_ (the Dvm component) to be completely dissolved in Bdm in a bulk pyrolytic composition.

The most convincing evidence for low *χ*_Ca_ at high temperatures is the coexistence of Bdm and Dvm in the run product with the Ca4Fe10 bulk composition at 40 GPa and 2,600 K (Fig. 2). Two perovskites are clearly observed in the STEM-EDS mapping (Fig. 2a). Therefore, the *χ*_Ca_ must be less than 0.04 p.f.u. under these conditions.

## Chemical equilibrium between bridgmanite and davemaoite

The low Ca content in Bdm observed in our study is unlikely to result from residual low-temperature Bdm and metastable persistence at high temperatures due to sluggish kinetics^15^ (Supplementary Text). The following evidence suggests chemical equilibrium has been reached in our runs. First, the phase compositions in the Ca4Fe10 and Ca8Fe10 samples are essentially identical. Second, the dihedral angles of approximately 120° at the triple junction of grains indicate a textural equilibrium. Third, the compositions of Bdm are nearly homogeneous within each grain (Fig. 1).

**Extended Data Fig. 4:**
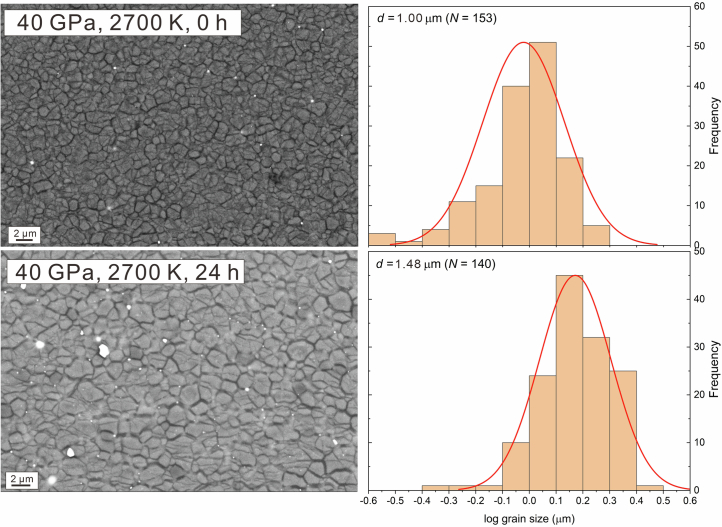
SEM images of run products with Ca8Fe10 bulk composition formed under 40 GPa and 2700 K and their grain size distributions. The top and bottom ones are the zero-time run and run with 24 h annealing time.

To further resolve the possible kinetic issue, a zero-time run was conducted at 40 GPa and 2,700 K using the Ca8Fe10 samples to confirm that chemical equilibrium has been reached in our annealing runs. The grain size in the zero-time run and the run annealed for 24 h was found to be 1.00 μm and 1.48 μm, respectively (Extended Data Fig. 4). Accordingly, a 240 nm rim of Bdm grew during annealing at 2,700 K. The composition of these rim parts should represent the composition of Bdm formed at 2,700 K. The low Ca content in these parts (Fig. 1b, Ca p.f.u. ≤ 0.021) indicates that the Ca content in Bdm formed under these conditions is low. More important, the comparable Ca content in the rim and core parts (Fig. 1) indicates that Ca diffusion is fast enough to achieve equilibrium under these conditions.

The chemical equilibrium between Bdm and Dvm is also supported by previous diffusion data. The self-diffusivity of Mg in Bdm is 6.3 × 10^–17^ m^2^ s^–1^ at 25 GPa and 2,700 K (ref. ^20^). This leads to a diffusion length of 2.2 μm at these conditions for 24 h. Even if the diffusion coefficient were reduced to 1/100 by assuming the activation volume of 7 cm^3^ mol^–1^, which is larger than the activation volume of Mg diffusion in silicate (for example, 5 cm^3^ mol^–1^ for olivine^21^), we should be able to observe diffusion profiles of ~100–200 nm at 40 GPa. Nevertheless, our run product synthesized at 2,700 K and ~40–50 GPa for 24 h did not show any chemical variation analogous to a diffusion profile exceeding 100 nm in length.

## Two perovskites exist in the lower mantle

**Fig. 3:**
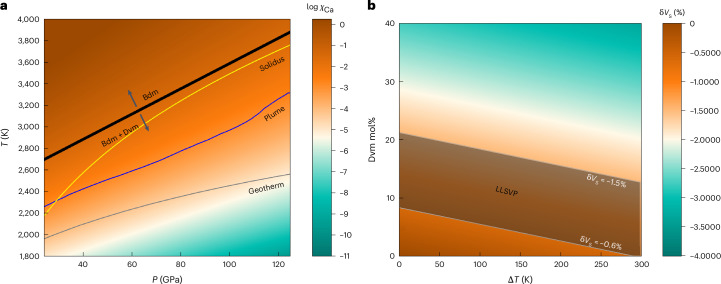
*χ*_Ca_ in bridgmanite under the lower-mantle-relevant conditions and the calculated seismic anomaly caused by a Dvm-enriched region. **a**, The *χ*_Ca_ in bridgmanite under the lower-mantle-relevant conditions. The black line represents the *χ*_Ca_ = 0.08. Dvm and Bdm should coexist in the area lower than this line. The grey, purple and yellow lines represent mantle geotherm^22^, plume geotherm^23^ and solidus of pyrolite^24^, respectively. **b**, The S-wave anomaly is calculated as a function of Dvm content in the Dvm-enriched domains and the excess temperature of this domain relative to the surrounding mantle at 120 GPa. The darkly shaded area represents conditions that align with the LLSVP seismic anomaly, with δ*V*_S_ in the –0.6% to –1.5% range^37^.

Thermodynamic extrapolation of the Ca solubility data down to the core–mantle boundary conditions indicates that Dvm exists throughout the lower mantle (Fig. 3a and Methods). With a pyrolitic bulk composition containing 3.21 mol% CaO, a Bdm with *χ*_Ca_ of 0.08 p.f.u. would consume all the available Ca. Our model, however, suggests that the equilibrium *χ*_Ca_ is much smaller than 0.08 along the mantle geotherm^22^ and plume geotherm^23^. The temperature required to form a single perovskite in the lower mantle even exceeds the solidus of pyrolite^24^ by 100 K. Therefore, Dvm is expected to persist in the lower mantle for the majority of geological time, even though the ancient mantle is considerably hotter than the present mantle^25^.

The existence of Dvm in the solid lower mantle over geological time has substantial implications for geochemistry and geophysics. Since Dvm is the last phase to solidify from a peridotitic melt^7–9^, it will be a major crystallizing phase during the late stages of the basal magma ocean^26^. A cumulate layer enriched in Dvm should have formed. The subducted slabs may have swept this Dvm-enriched layer to form Dvm-enriched domains^27^ beneath Africa and the Pacific Ocean. These domains may include distinct materials subducted during different periods, which can explain the chemical dichotomy between the African and Pacific mantle domains^28^ and between the Northern and Southern Hemispheres^29^. In addition, as Dvm can contain trace elements such as rare-earth elements, uranium and thorium, these Dvm-enriched domains at the bottom of the lower mantle could serve as reservoirs for the HIMU component of ocean island basalts^4^. The enrichment of radioactive isotopes, such as ^238^U, in Dvm in the early stage of Earth’s history could result in the preservation of high temperatures within the core, which may have contributed to the delayed formation of the inner core^30^.

The Dvm-enriched domains at the base of the lower mantle may also suggest an interpretation of the origin of the LLSVPs^31^. The relatively sharp boundaries of the LLSVPs with the surrounding high-velocity regions suggest that the LLSVPs have a distinct composition from the high-velocity regions^32^. The long stability of the LLSVP structure also suggests that the LLSVPs have a different composition from the ambient mantle^33^. This compositional heterogeneity may stem partly from these Dvm-enriched domains. Some researchers have also proposed that subducted Dvm-rich oceanic crust might be partially separated from the slab during the lateral D” flow towards the LLSVP margins and is likely to accumulate on top of the cumulate-dominated base of the LLSVPs^27,34^, further increasing the Dvm content in the LLSVPs.

The Dvm amount in these domains required to account for the seismic anomaly of the LLSVPs is estimated to be less than 20 mol%. Assuming that the material surrounding the LLSVPs is Bdm, as suggested by the low Mg/Si ratio in the lower mantle^35,36^, and that the LLSVPs consist of Bdm and Dvm, our calculations (Methods) suggest that at 120 GPa (2,600 km depth), ~8–20 mol% Dvm is sufficient to reproduce the –0.6% to –1.5% shear-wave-velocity anomaly observed in the LLSVPs^37^ if the LLSVPs have the same temperature as the surrounding materials (Fig. 3b). LLSVPs hotter than the surrounding materials decrease the proportion of Dvm required to account for the LLSVPs seismic anomaly (Fig. 3b). In the case that the surrounding materials contain post-perovskite, the required Dvm amount is reduced because post-perovskite has a higher shear-wave velocity than Bdm^38^. Considering that Bdm in the LLSVPs is Fe rich^7^, the amount of Dvm required to explain the LLSVPs’ seismic anomaly may be even smaller because Fe decreases the S-wave velocity^39,40^.

This amount of Dvm is supported by the crystallization experiments from silicate melt. The melting phase diagram in the MgSiO_3_–CaSiO_3_ system at a pressure of 24 GPa shows a eutectic point with a MgSiO_3_–CaSiO_3_ ratio of 4/1, that is, a Dvm fraction of 20 mol% (ref. ^41^). Although the Dvm amount crystallized from a pyrolitic melt may be less than 20 mol% during the basal magma ocean solidification, it must be greater than the Dvm amount in the pyrolite (~7 mol%). This is because the Ca content in the residual melt increases during solidification^7^. Therefore, this Dvm-enriched layer provides an explanation for the origin of the LLSVPs. However, further experiments on the velocities of Bdm and Dvm and the melting relationships in a more complex system under lowermost mantle conditions are needed to better constrain the proportion of Dvm in the LLSVPs.

## Methods

### Starting material preparation

Glasses with the target compositions of (1) Ca_0.5_Mg_0.5_SiO_3_ (Ca50), (2) Ca_0.04_Fe_0.1_Mg_0.86_SiO_3_ (Ca4Fe10), (3) Ca_0.08_Fe_0.1_Mg_0.82_SiO_3_ (Ca8Fe10), (4) Ca_0.08_Fe_0.11_Al_0.11_Mg_0.7_SiO_3.1_ (Fe11Al11) and (5) Ca_0.5_Fe_0.2_Al_0.2_Mg_0.3_Si_0.8_O_3_ (Fe20Al20) were prepared by melting mixtures of MgO, SiO_2_, CaO and Fe_2_O_3_ powders in the prescribed ratio at 1,923 K using a high-temperature furnace, followed by rapid quenching in cold water. The compositions of the synthesized glasses were confirmed using an energy-dispersive X-ray spectrometer equipped with a LEO1530 scanning electron microscope at the Bayerisches Geoinstitut (BGI), Universität Bayreuth and are reported in Extended Data Table 1. The composition and its standard deviations for each glass were determined by averaging several point analyses of the glass.

### Multi-anvil experiments

High-pressure–temperature experiments were carried out using a 15-MN ultrahigh-pressure multi-anvil press (IRIS-15) equipped with the Osugi-type guide-block system at the BGI^18^. The pressure and temperature ranges of the experiments were from 27 to 50 GPa and from 2,300 to 2,700 K, respectively (Extended Data Table 2). The BGI standard 7/3 assembly was used in the 27 GPa experiments, while 5.7/1.5 assemblies were used in 33‒50 GPa experiments (Supplementary Fig. 1). For the 27 GPa experiment, a trace amount of Mg(OH)_2_ was put at the bottom of the welded platinum (Pt) capsules. The release of H_2_O from Mg(OH)_2_ served as a flux to promote the grain growth. Large grain sizes can avoid the overlapping effect from the nearby grain during the post-experiment composition measurement. Although a small quantity of H_2_O may be incorporated in Bdm, any trace component does not affect the equilibrium of the major components due to Raoult’s law. In the runs at 33–40 GPa and 2,300 K, two starting materials were loaded into capsules made of Pt foil with outer and inner diameters of 0.6 and 0.5 mm. These capsules were then loaded into an Al_2_O_3_ sleeve with outer and inner diameters of 1.0 and 0.6 mm, respectively, within a LaCrO_3_ heater in a 5 wt% Cr_2_O_3_‐doped MgO octahedron with an edge length of 5.7 mm. In the runs at 40–50 GPa and 2,600–2,700 K, two starting materials were loaded into capsules made from rhenium foil with outer and inner diameters of 0.5 and 0.4 mm, except for run I1369, in which a Pt capsule was used for the Ca50 sample and no metal capsule was used for the Fe20Al20 sample. These capsules were then loaded into an Al_2_O_3_ sleeve with outer and inner diameters of 0.8 and 0.5 mm within a LaCrO_3_ heater. ZrO_2_ sleeves were used as thermal insulators outside the LaCrO_3_ heater in these high-temperature runs.

A type-D thermocouple was inserted through the heater, and its junction was sandwiched between two rhenium capsules to monitor the sample temperature. The sample assembly was compressed to target pressures at ambient temperature using eight tapered tungsten carbide anvils with an edge length of 26 mm and a truncation of 3 mm or 1.5 mm (ref. ^18^). After reaching the target pressure, the sample was heated to the target temperature at a ramp rate of 200 K min^–1^. The power–temperature relationships of runs at 40 GPa are shown in Supplementary Fig. 2, demonstrating a consistent heating record across the different runs. After reaching the target temperature, the sample was annealed for 24 hours. Experiments I1249 and I1459 were run on thermocouple-control mode for the entire 24 hours. Other experiments were controlled by power. The fluctuation of power in all runs was less than 2 W, and the fluctuation of temperature in I1249 and I1459 was less than 10 K during annealing. The fluctuation of oil pressure in all runs was less than 0.003 MN during annealing. The samples were then quenched to room temperature by turning off the heater and decompressed to ambient pressure for more than 16 hours. For the zero-time run, the sample was immediately quenched after reaching the target temperature.

### Chemical and XRD analysis

The run products were mounted in epoxy resin. Their cross sections were prepared by polishing them with sandpaper and diamond pastes. The run products were then examined using a scanning electron microscope, and the grain sizes of I1471 and 1466 were measured (Extended Data Fig. 4). Lamellae of 100–200 nm thickness were cut using a focused ion beam facility at BGI for all the recovered samples. The chemical mappings of the lamellae were obtained using a scanning transmission electron microscope (FEI Titan G2 80-200 S/TEM) equipped with an energy-dispersive X-ray spectrometer (four silicon drift detectors, Bruker Quantax) operating at 200 kV. The EDS maps were acquired with a resolution of 10–15 nm per pixel and a dwell time of 16 μs using a sub-nanometre electron beam with less than 0.08 nA probe current. The total acquisition time was 60–90 minutes to accumulate statistically enough characteristic X-ray counts in a quantitative EDS map. During acquisition, an image drift correction function was always activated to avoid artefacts in the map. To obtain quantitative compositions of the samples, we corrected for *Z* number and absorption effects in the evaluations of the EDS spectra^42,43^. The absorption effect was corrected by changing the input lamellae thickness so that the O mol% in Bdm and Dvm are 60. For accurate quantification based on the *Z*-number correction, a natural pyrope–almandine garnet (Prp73Alm17Grs11 (ref. ^44^)) was used as an EDS standard for elements of Mg, Al, Si, Ca and Fe. Composition and its standard deviations for each sample were determined by averaging several area analyses of the sample. We note that the determined Dvm composition may have bigger analytical error than Bdm, as documented in the higher than 1 of Si p.f.u in their stoichiometry. This may be caused by the selective Mg diffusion during electron beam irradiation in amorphous materials^45^.

The Fe^3+^/ΣFe ratios of Bdm samples of I1268, I1459 and I1510 were evaluated by electron-energy-loss spectroscopy. The Fe-*L*_*2,3*_ edge electron energy-loss near-edge structure was acquired in STEM mode in a dual-mode EELS spectrometer (Gatan energy filter Quantum SE). The energy resolution is about 0.8 eV in the corresponding zero-loss spectra. The Fe-*L*_*2,3*_ energy-loss near-edge structure was quantified according to the procedure described in ref. ^46^. The Fe^3+^/ΣFe ratios were calculated by using a universal calibration curve^47^ and the fitting program EELSA provided by Clemens Prescher.

The compositions of the run products in the Ca50 sample at 27 GPa and 2,300 K were also analysed with a JEOL JXA-8200 electron microprobe (EPMA), operating at 15 kV and 5 nA with enstatite as standards for Mg and Si and diopside as a standard for Ca.

Phases in the recovered samples were identified using a micro-focused X-ray diffractometer (Bruker AXS D8 Discover) equipped with a two-dimensional solid-state detector and a micro-focus source of Co–K*α* radiation operated at 40 kV and 500 µA at the BGI. The exposure time was over 1 hour for each sample with a beam size of ~100 µm.

### Fitting of experimental data

The *χ*_Ca_ reported in this study were fitted to the following exponential equation:

1 $${\chi }_{{\rm{Ca}}}=\exp\left({{A}}+\frac{{{B}}}{T}+{{C}}\times \frac{P}{T}\right)$$

where *A*, *B* and *C* are fitted parameters; *P* and *T* are pressure and temperature. We ignore the composition effect on the fitting because it is negligible as suggested by our experiments. At equilibrium, *χ*_Ca_ is determined by the following reaction

2 $${{\rm{CaSiO}}}_{3}\left({\rm{Dvm}}\right)={\rm{CaSi}}{{\rm{O}}}_{3}({\rm{Bdm}})$$

The standard Gibbs free energy change for this reaction, Δ*G*^0^, can be expressed as

3 $$-\Delta {{{G}}}^{0}={RT}\,\mathrm{ln}\frac{{a}_{{{\mathrm{CaSi}}}{{\mathrm{O}}}_{3}}^{{{\mathrm{Bdm}}}}}{{a}_{{{\mathrm{CaSi}}}{{\mathrm{O}}}_{3}}^{{{\mathrm{Dvm}}}}}$$

where ${a}_{{{\rm{CaSiO}}}_{3}}^{{\rm{Bdm}}}$ and ${a}_{{{\rm{CaSiO}}}_{3}}^{{\rm{Dvm}}}$ are the activity of CaSiO_3_ in the Bdm and Dvm, respectively; *R* is the gas constant. Δ*G* can be expressed as

4 $$\Delta {{{G}}}^{0}=\Delta {{{H}}}^{1{\rm{bar}}}-T\Delta {S}^{1{\rm{bar}}}+P\Delta V$$

where Δ*H*^1bar^ and Δ*S*^1bar^ are the standard enthalpy and entropy change at 1 bar for reaction (2); Δ*V* is the volume change for reaction (2). We take ${a}_{{{\rm{CaSiO}}}_{3}}^{{\rm{Dvm}}}=1$ and ${a}_{{{\rm{CaSiO}}}_{3}}^{{\rm{Bdm}}}$ as the CaSiO_3_ mole fraction in Bdm, namely, *χ*_Ca_. Combing equations (3) and (4), we have

5 $${\chi }_{{\rm{Ca}}}=\exp\left(-\frac{\Delta {S}^{1{\rm{bar}}}}{R}+\frac{\Delta {{{H}}}^{1{\rm{bar}}}}{{RT}}+\Delta {{V}}\times \frac{P}{{RT}}\right)$$

namely, equation (1). The best fitting yields *A* = 12.3, *B* = –36,560 and *C* = –171. The negative *B* and *C* values confirm the positive temperature and negative pressure dependence of *χ*_Ca_.

Our fitting indicates that Dvm should coexist with Bdm even at the pyrolite solidus temperatures. The *χ*_Ca_ at 50, 80 and 120 GPa are 0.019, 0.041 and 0.046 under the solidus temperature. Because these *χ*_Ca_ are smaller than the Ca content in the pyrolite (0.08 p.f.u.), the Dvm should always exist even under the pyrolite solidus temperatures.

### Modelling of rock seismic velocities

The shear-wave velocity (*V*_S_) was calculated for a mixture of Bdm and Dvm (Dvm-enriched domain) and pure Bdm (surrounding mantle) using the BurnMan software package^48^ with the thermodynamic parameters from ref. ^49^. The Bdm contains 80 mol% MgSiO_3_, 5 mol% FeSiO_3_ and 15 mol% FeAlO_3_ to match the expected Bdm composition in pyrolite^50^. We assumed that Bdm in the Dvm-enriched domains has the same composition as Bdm in the surrounding mantle and varied the Dvm content and excess temperature of the domains. The calculated shear-wave-velocity anomalies are shown in Fig. 3 by different colours.

## Online content

Any methods, additional references, Nature Portfolio reporting summaries, source data, extended data, supplementary information, acknowledgements, peer review information; details of author contributions and competing interests; and statements of data and code availability are available at 10.1038/s41561-025-01657-9.

## Supplementary information

Supplementary Information Supplementary Figs. 1–5 and text.

## Figures and Tables

**Extended Data Table 1:**
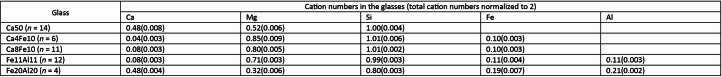
Compositions of synthesized starting materials

Standard deviations (1σ precision) are listed in the parentheses. *n* is the number of measured areas.

**Extended Data Table 2:**
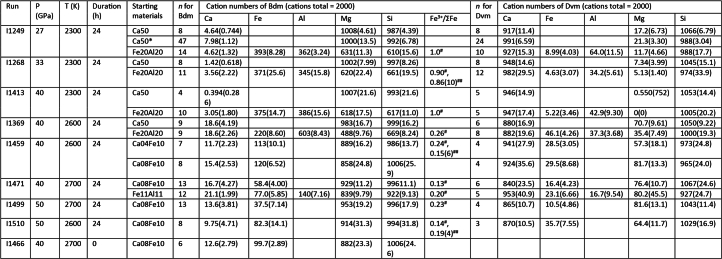
Experimental conditions and compositions of the phases in the run products with total cations number normalized to 2000

Standard deviations (1σ precision) are listed in the parentheses. *n* is the number of measured areas (for TEM measurements) or points (for EPMA measurements).

*The measurement was done by EPMA.

# The Fe^3+^/ΣFe was calculated by charge balance assuming 3000 oxygen in Bdm.

## The Fe^3+^/ΣFe was calculated using electron-energy-loss spectroscopy.

## Data Availability

The data that support the findings of this study are provided in Extended Data Tables 1 and 2 and are available via figshare at 10.6084/m9.figshare.27682386 (ref. ^51^).
