# Supplementary material for: Persistence of davemaoite at lower-mantle conditions
Source: Nat Geosci. 2025 Feb 28;18(4):365–9. doi: 10.1038/s41561-025-01657-9 (PMC11981924; doi:10.1038/s41561-025-01657-9)
Supplement: Supplementary file 1 — Supplementary Figs. 1–5 and text. [file 41561_2025_1657_MOESM1_ESM.pdf]

nature geoscience

Supplementary information https://doi.org/10.1038/s41561-025-01657-9

Persistence of davemaoite at lower-mantle conditions

In the format provided by the authors and unedited

The PDF file includes:

Supplementary Text
Supplementary Figures 1 – 5

Supplementary Text

Ca-rich bridgmanite as a metastable phase

Ko et al.[1] synthesized a Ca-rich Bdm (Ca p.f.u. >0.08) under conditions above 40 GPa and 2300 K. They concluded the absence of Dvm in the hot regions in the deep lower mantle. However, we think that this phase may be metastable, as demonstrated by previous studies[2, 3, 4]. Funamori et al.[2] conducted LH-DAC experiments on a MORB glass at 2250 K, 2750 K, and 3250 K. The results indicated that the presence of a single Ca-Bdm phase in the lowest-temperature run, while the higher-temperature runs exhibited the coexistence of Bdm and Dvm phases. Asahara et al.[3] showed that a (Mg,Fe,Al)SiO_3 glass was initially transformed to Ca-Bdm at 1600 K and then decomposed to Bdm with 2 mol.% of $CaSiO_3$ and Dvm at 2100 K. Sano et al.[4] showed that Ca-Bdm was initially formed from a MORB glass at 1400 K and then decomposed to Bdm with a $CaSiO_3$ content of 3 mol.% and Dvm at 1900 K. These three studies demonstrated that a single phase of Ca-Bdm, rather than two perovskites, was formed at relatively low temperatures and decomposed to Bdm and Dvm at higher temperatures. Given that Ca-Bdm should have a stability field at higher temperatures than Bdm + Dvm[5], the Ca-rich Bdm observed in these studies was a metastable phase. The discrepancy between Ko et al.[1] and these three studies cannot be attributed to the difference in composition, as we have already shown that the compositional effects are negligible. Indeed, Ko et al.[1] also observed the formation of Bdm + Dvm at their highest temperatures. They attributed the presence of Bdm + Dvm to melting: a $CaSiO_3$-rich melt migrated into the lower-temperature regions of their sample chambers due to the temperature gradient and was subsequently crystallized into Dvm. However, once the melt has formed, it should remain in the high-temperature area, as a number of high-pressure experiments have demonstrated[6].

The high Ca content in Bdm in the runs where Bdm and Dvm both appear at highest temperatures in Ko et al.[1] may be attributed to a kinetic effect. Sano et al.[4] observed that the Ca content in Bdm is 3 mol % at 1900 K following the decomposition of Ca-rich Bdm. This value is considerably higher than that observed in previous studies under comparable conditions [7, 8]. The high Ca content in Bdm may be inherited from previously formed Ca-rich Bdm and their assembly may not reach chemical equilibrium at 1900 K. This indicates that once the Ca-rich Bdm forms, even after it decomposes into Bdm at a higher temperature, the resulting Bdm may still have a high Ca content due to kinetic issues. Therefore, although Ca-rich Bdm was successfully synthesized in Ko et al.[1], it may be a metastable phase and should not exist in the Earth's lower mantle.

Comparison with previous studies

There are some laser heater-diamond anvil cell (LH-DAC) experiments reporting the absence of Dvm diffraction peaks[1, 9, 10, 11] or the absence of Dvm from the recovered sample by scanning transmission electron microscopy (STEM) observation[1, 10, 12]. However, the absence of Dvm diffraction peaks does not indicate the absence of Dvm. It could be due to weak diffraction or overlap with Bdm[9]. Ref[11] found only Bdm diffraction peaks from a pyrolite starting material under lower mantle conditions. However, ferropericlase (Fper) must be present under such conditions. As Dvm and Fper, respectively, comprise 7 and 17 vol.% of pyrolite, the absence of Fper suggests that the presence of Dvm may also have been overlooked. The absence of Dvm by STEM observation does not indicate the absence of Dvm, either. The observed area may not have contained Dvm due to the small TEM sample. The recovered sample from Ref[12] does not contain Dvm in the STEM analysis, but the Ca content in their Bdm (1.1 wt %) is lower than the starting pyrolite glass (3.8 wt %). This indicates the presence of Dvm in the run

products. In fact, Ref[13] found Dvm diffraction peaks in situ in the same sample used in Ref[12]. Therefore, the absence of Dvm XRD peaks or STEM observation cannot rule out the possibility of the coexistence of Bdm and Dvm.

The slightly positive temperature dependence of χ_{Ca} in Bdm is supported by previous studies. χ_{Ca} in $MgSiO_3$ Bdm increases by only 0.005 p.f.u. from 1773 K to 2173 K at 27 GPa[7]. χ_{Ca} in a Fe- and Al-bearing Bdm also increased by 0.005 from 1273 to 2073 K at 32 GPa[3]. Ref[1], however, reported a more than 1.5 orders of magnitude higher increasing rate in Ca content from 2073 to 2284 K at pressures higher than 40 GPa. Here, although our results suggest a slightly high increasing rate (0.004 p.f.u./100 K at pressures higher than 40 GPa), it is still much smaller than in Ref[1].

The negative pressure dependence of χ_{Ca} in Bdm is also supported by previous studies. χ_{Ca} decreases by more than half at 2300 K from 104 GPa to 111 GPa[12]. In the pyrolitic bulk composition, χ_{Ca} decreases by 0.006 from 43 to 47 GPa, although temperature increases by 100 K at the same time[8]. Recent melting experiments[14] found that the Ca content in Bdm near the liquidus of pyrolite remains almost the same, indicating a negative pressure dependence. A theoretical study also suggested a negative pressure dependence of χ_{Ca} in $MgSiO_3$ Bdm[15].

The small effect of Fe on χ_{Ca} found in this study is consistent with some previous studies. No Fe effect was found at 32 GPa and approximately 2200 K[16]. Ref[17] reported a 0.006 p.f.u. increase in χ_{Ca} by an increase of 0.1 p.f.u. of Fe in Bdm, indicating a small effect of Fe. These studies, together with our results, contradict the observation that adding Fe can substantially increase χ_{Ca}[1]. Fe^{3+} is found to increase the χ_{Ca}, but the magnitude is much smaller than the observation of Ref[10].

The χ_{Ca} in this study is generally lower than in previous studies under similar conditions[7]. They reported Ca p.f.u. in Bdm as 0.016(3) at 2200 K and 27 GPa for $MgSiO_3$ Bdm, while our result is 0.005(1) at the same pressure but 100 K higher temperature. One possible reason is the different analytical methods used in these two studies. They used EPMA while we used STEM. In order to test this hypothesis, we also measured our Ca50 sample at 27 GPa and 2300 K using EPMA. The measured results show a 0.08(1) Ca p.f.u. in Bdm (Extended Data Table 2), which is slightly higher than the STEM results. The higher value in Ref[7] could be due to the overlap of Dvm and Bdm grains during the measurement caused by their same grain size. Although they did not report the grain size, their heating time is only 5 min. So the grain size was probably very small compared with ours. Even if we would have applied a correction factor of 0.08/0.05 = 1.6 to the Ca-content of our bridgmanite analyses, the resulting maximun χ_{Ca} of 0.3 would correspond to only 1.68 mol % CaO, still smaller than the 3.21 mol% CaO in a bulk pyrolitic composition.

Other studies using STEM also give higher Ca contents [1, 3, 10, 12]. This could be due to the small grain sizes in these studies hindering the resolution of STEM-EDS. The grain size in our sample is greater than 1μm, while it is less than 400 nm in these studies. As the resolution of STEM is determined by the lamella thickness, the measured EDS signals are affected by the surrounding grains when the grain size is smaller or comparable to the lamella thickness. Indeed, a 0.015 Ca p.f.u. in Bdm at 2100 K, 32 GPa with a grain size of 400 nm was reported[3], while a 0.05 Ca p.f.u. at the same temperature but 82 GPa was reported with a Bdm grain size of less than 200 nm[12]. As pressure suppresses χ_{Ca}, the larger value in Ref[12] cannot be explained by the higher pressure. The grain sizes in Ref[1] and Ref[10] are ~100 nm and cannot be resolved, which may be the reason for their apparent high χ_{Ca}. The idea that the smaller grain size in these studies could affect the Ca content measurement is also supported by the larger analytical error in these studies compared with ours.

The χ_{Ca} could become very high at a temperature close to the solidus. A 0.03-0.05 Ca p.f.u. in Bdm was reported at a temperature of 20 K below the solidus at 24 GPa[18] and 56 GPa and ~2800 K[19]. However, higher pressure experiments suggested that χ_{Ca} is less than 0.025 p.f.u. at pressures from 58 to 135 GPa at the pyrolite solidus[20, 21]. Therefore, with the negative effect of pressure, the χ_{Ca} is still lower at high pressures even close to the pyrolite solidus.

Nevertheless, even at the highest χ_{Ca} reported in the literature (0.05 p.f.u.), it is still lower than the Ca content in pyrolite, demonstrating that two perovskites should exist in a pyrolite lower mantle.

Composition of davemaoite

$MgSiO_3$ is the most abundant minor component in Dvm. The solubility of Mg in Dvm (χ_{Mg}) is much higher than χ_{Ca} in Bdm. χ_{Mg} can be up to 0.08 p.f.u. at 2600 K and 40 GPa, while the highest χ_{Ca} is 0.01 at the same conditions. Temperature has a strong effect on χ_{Mg} (Supplementary Figure 3A). For the Ca50 and Fe20Al20 samples, χ_{Mg} increases by 0.07 and 0.04 p.f.u. from 2300 K to 2600 K at 40 GPa, respectively. Pressure has a negative effect on χ_{Mg} for the sample annealed at a temperature of 2300 and 2600 K but a negligible effect on the sample annealed at a temperature of 2700 K (Supplementary Figure 3B).

The intersolubility of Bdm and Dvm is show in the Supplementary Figure 4. It is consistent with the low temperature solvus from previous multi-anvil study[7]. It also agrees with the ab initio atomistic simulation in the $MgSiO_3$-$CaSiO_3$ system[15], although their Mg solubility in Dvm is more restricted.

The Fe and Al contents in Dvm are limited (Extended Data Table 2). The partition coefficient of Fe between Bdm and Dvm at 2300 K is always higher than 40 but decreases to approximately 3 at temperatures above 2600 K (Supplementary Figure 5). This could be because most of Fe in Bdm is Fe^{3+} at 2300 K but is Fe^{2+} at 2600 K. This indicates that although Fe^{2+} prefers Bdm, the extent is much smaller than Fe^{3+}. The partition coefficient of Al between Bdm and Dvm ranges from 6 to 10, indicating that Al also prefers Bdm. Therefore, Bdm is the main host for Fe and Al in the lower mantle.

Reference

1. Ko B, Greenberg E, Prakapenka V, Alp EE, Bi W, Meng Y*, et al.* Calcium dissolution in bridgmanite in the Earth's deep mantle. *Nature* 2022, **611**(7934)**:** 88-92.

2. Funamori N, Jeanloz R, Miyajima N, Fujino K. Mineral assemblages of basalt in the lower mantle. *Journal of Geophysical Research: Solid Earth* 2000, **105**(B11)**:** 26037-26043.

3. Asahara Y, Ohtani E, Kondo T, Kubo T, Miyajima N, Nagase T*, et al.* Formation of metastable cubic-perovskite in high-pressure phase transformation of Ca (Mg, Fe, Al) Si_2O_6. *American Mineralogist* 2005, **90**(2-3)**:** 457-462.

4. Sano A, Ohtani E, Litasov K, Kubo T, Hosoya T, Funakoshi K*, et al.* In situ X-ray diffraction study of the effect of water on the garnet–perovskite transformation in MORB and implications for the penetration of oceanic crust into the lower mantle. *Physics of the Earth and Planetary Interiors* 2006, **159**(1-2)**:** 118-126.

5. Muir JM, Thomson AR, Zhang F. The miscibility of calcium silicate perovskite and bridgmanite: A single perovskite solid solution in hot, iron-rich regions. *Earth and Planetary Science Letters* 2021, **566:** 116973.

6. Nomura R, Hirose K, Uesugi K, Ohishi Y, Tsuchiyama A, Miyake A*, et al.* Low core-mantle boundary temperature inferred from the solidus of pyrolite. *Science* 2014, **343**(6170)**:** 522-525.

7. Irifune T, Miyashita M, Inoue T, Ando J, Funakoshi K, Utsumi W. High-pressure phase transformation in CaMgSi2O6 and implications for origin of ultra-deep diamond inclusions. *Geophysical Research Letters* 2000, **27**(21)**:** 3541-3544.

8. Irifune T, Shinmei T, McCammon CA, Miyajima N, Rubie DC, Frost DJ. Iron partitioning and density changes of pyrolite in Earth's lower mantle. *Science* 2010, **327**(5962)**:** 193-195.

9. Lee KK, O'Neill B, Panero WR, Shim S-H, Benedetti LR, Jeanloz R. Equations of state of the high-pressure phases of a natural peridotite and implications for the Earth's lower mantle. *Earth and Planetary Science Letters* 2004, **223**(3-4)**:** 381-393.

10. Creasy N, Girard J, Eckert Jr JO, Lee KK. The role of redox on bridgmanite crystal chemistry and calcium speciation in the lower mantle. *Journal of Geophysical Research: Solid Earth* 2020, **125**(10)**:** e2020JB020783.

11. Ono S, Ohishi Y, Isshiki M, Watanuki T. In situ X-ray observations of phase assemblages in peridotite and basalt compositions at lower mantle conditions: Implications for density of subducted oceanic plate. *Journal of Geophysical Research: Solid Earth* 2005, **110**(B2).

12. Sinmyo R, Hirose K. Iron partitioning in pyrolitic lower mantle. *Physics and Chemistry of Minerals* 2013, **40:** 107-113.

13. Sinmyo R, Hirose K, Muto S, Ohishi Y, Yasuhara A. The valence state and partitioning of iron in the Earth's lowermost mantle. *Journal of Geophysical Research: Solid Earth* 2011, **116**(B7).

14. Nabiei F, Badro J, Boukaré CÉ, Hébert C, Cantoni M, Borensztajn S*, et al.* Investigating magma ocean solidification on Earth through laser-heated diamond anvil cell experiments. *Geophysical Research Letters* 2021, **48**(12)**:** e2021GL092446.

15. Jung DY, Schmidt MW. Solid solution behaviour of $CaSiO_3$ and $MgSiO_3$ perovskites. *Physics and Chemistry of Minerals* 2011, **38:** 311-319.

16. Fujino K, Sasaki Y, Komori T, Ogawa H, Miyajima N, Sata N*, et al.* Approach to the mineralogy of the lower mantle by a combined method of a laser-heated diamond anvil cell experiment and analytical electron microscopy. *Physics of the Earth and Planetary Interiors* 2004, **143:** 215-221.

17. Corgne A, Liebske C, Wood BJ, Rubie DC, Frost DJ. Silicate perovskite-melt partitioning of trace elements and geochemical signature of a deep perovskitic reservoir. *Geochimica et Cosmochimica Acta* 2005, **69**(2)**:** 485-496.

18. Nomura R, Zhou Y, Irifune T. Melting phase relations in the $MgSiO_3$–$CaSiO_3$ system at 24 GPa. *Progress in Earth and Planetary Science* 2017, **4:** 1-11.

19. Lobanov SS, Holtgrewe N, Ito G, Badro J, Piet H, Nabiei F*, et al.* Blocked radiative heat transport in the hot pyrolitic lower mantle. *Earth and Planetary Science Letters* 2020, **537:** 116176.

20. Kesson S, Fitz Gerald J, Shelley J. Mineralogy and dynamics of a pyrolite lower mantle. *Nature* 1998, **393**(6682)**:** 252-255.

21. Kim T, Ko B, Greenberg E, Prakapenka V, Shim SH, Lee Y. Low melting temperature of anhydrous mantle materials at the core-mantle boundary. *Geophysical Research Letters* 2020, **47**(20)**:** e2020GL089345.

Figures

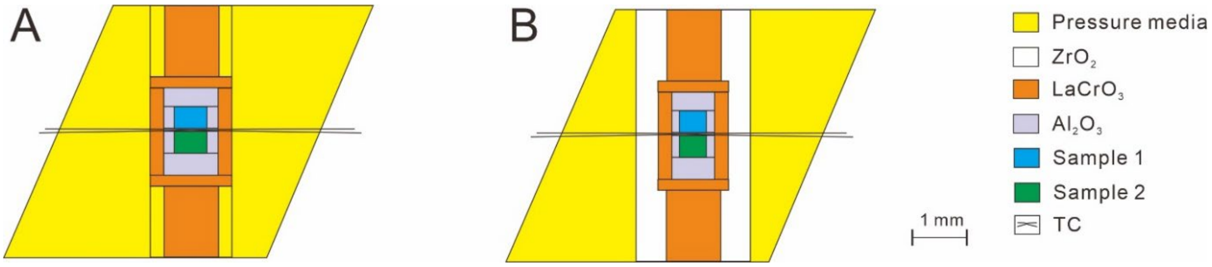

Supplementary Figure 1. 5.7/1.5 cell assemblies used in this study. (A) For experiments conducted at 2300 K. (B) For experiments conducted at temperature 2600 – 2700 K.

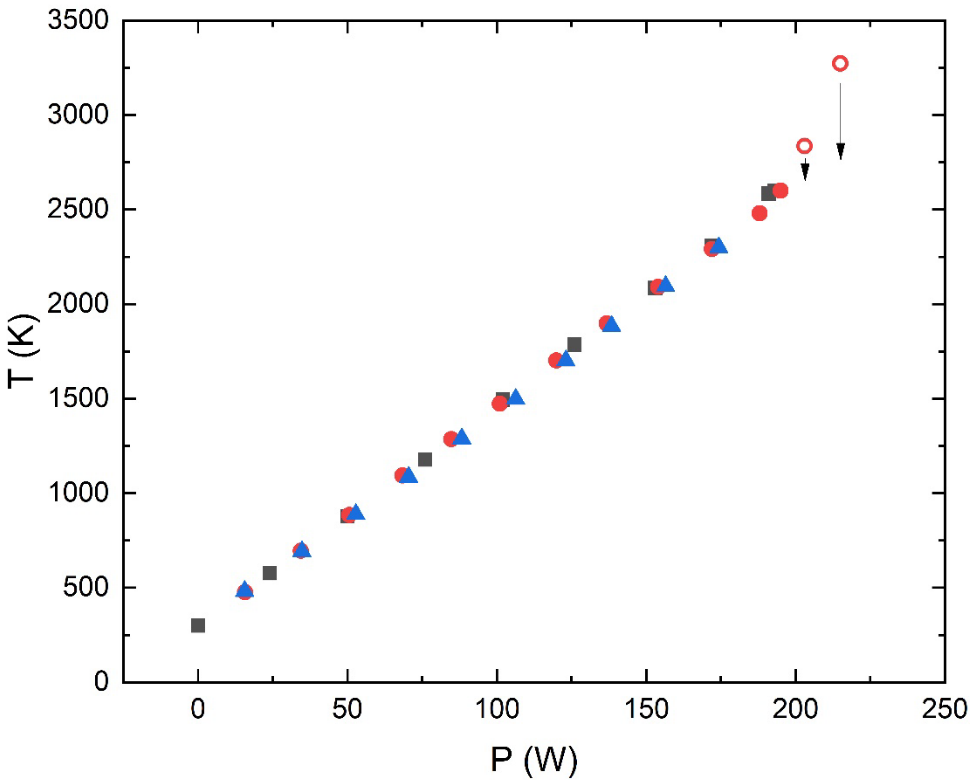

Supplementary Figure 2. Power-temperature relationship for runs conducted at 40 GPa. Black square, red circle and blue triangle represent run I1459, I1471 and I1392, respectively. The P-T relationship of I1471 deviates from the trend above 2600 K. We therefore used the P-T relationship to extrapolate to 2700 K. I1392 is another experiment conducted for another purpose but used the same cell assembly as the present one.

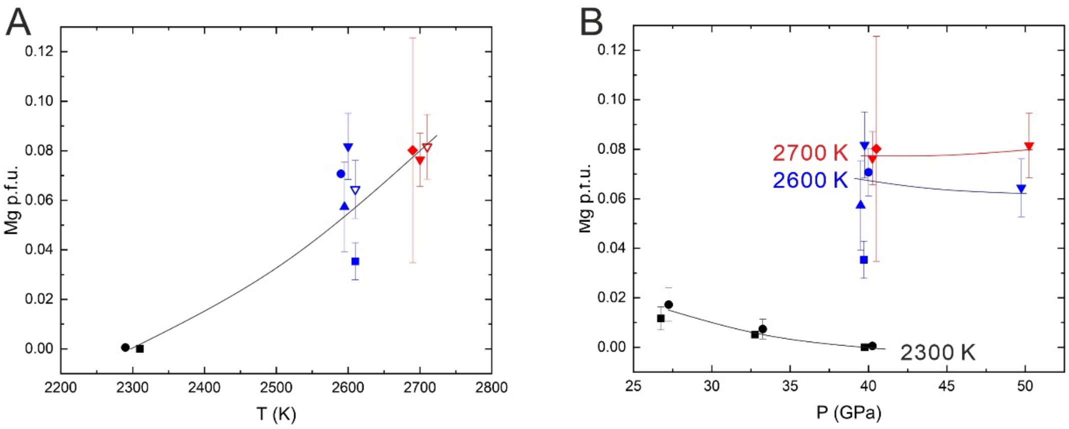

Supplementary Figure 3. Temperature (A) and pressure (B) dependence of χ_{Mg} in Dvm. The circle, triangle, inverted triangle, diamond and square symbols represent Dvm from Ca50, Ca4Fe10, Ca8Fe10, Fe11Al11 and Fe20Al20 samples, respectively. The black, blue and red colors represent Dvm synthesized at 2300, 2600 and 2700 K. The solid and open symbols in (A) represent 40 GPa and 50 GPa runs, respectively. The data points are offset slightly from their true X-values for better illustration. The lines are guides for eye. 2σ error bars are shown. The sample size (*n*) for each point is listed in the Extended Data Table 2.

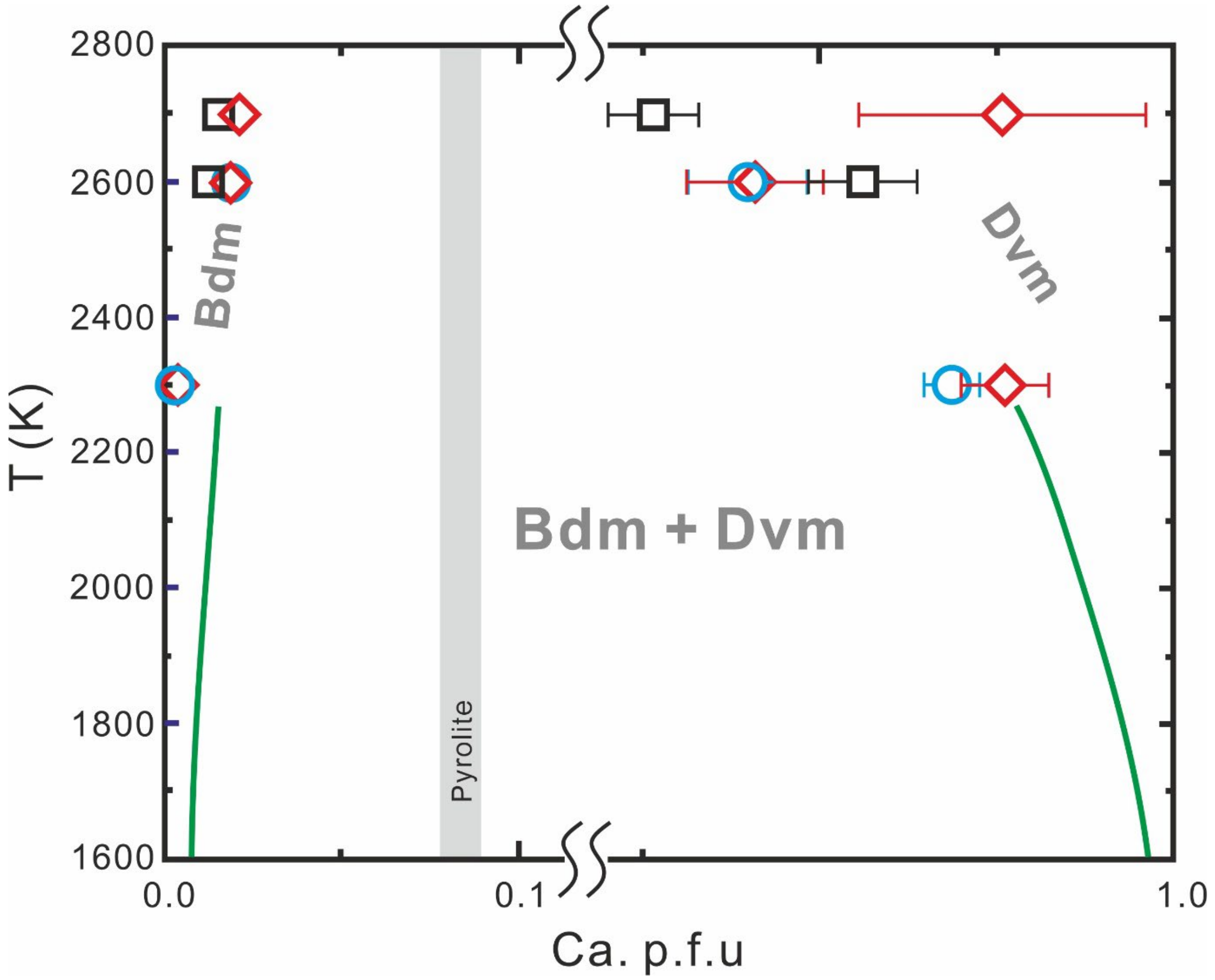

Supplementary Figure 4. Phase diagram of Bdm and Dvm. Each data point represents the average the Ca content in Bdm and Dvm from different pressures in the same/similar starting materials. CMS (blue cricle): Ca50 samples. CFMS (black square): Ca4Fe10 and Ca8Fe10 samples. CFMAS (red diamond): Fe11Al11 and Fe20Al20 samples. The green lines are the solvus between Bdm and Dvm from the previous study[7]. The shaded vertical line is the required value for Dvm fully dissolved in Bdm in a pyrolite composition. 2σ error bars are shown. The error bars for Bdm are smaller than the symbols' sizes. The sample size (*n*) for each point can be calculated from the Extended Data Table 2.

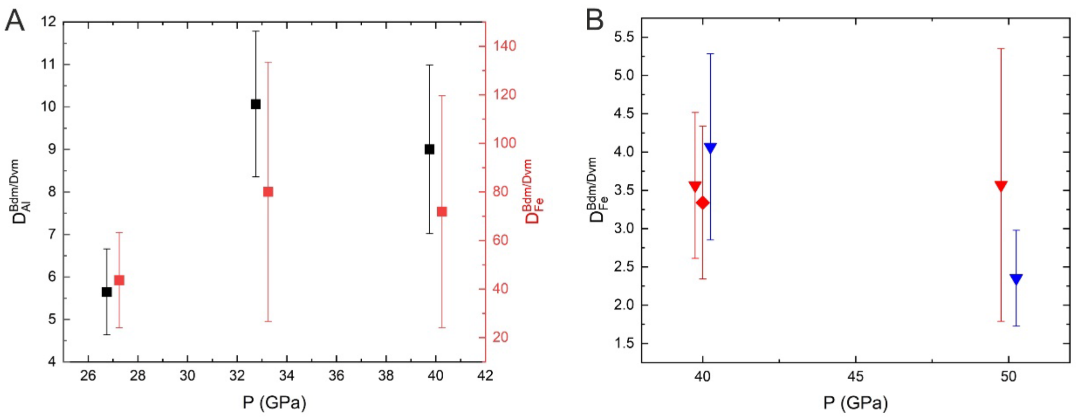

Supplementary Figure 5. Partition coefficients of Fe and Al between Bdm and Dvm. (A) Al (black) and Fe (red) partition coefficients between Bdm and Dvm at 2300 K in the Fe20Al20 sample. (B) Fe partition coefficients between Bdm and Dvm at 2600 – 2700 K. The inverted triangle and diamond symbols represent results from Ca8Fe10 and Fe11Al11 sample, respectively. The blue and red symbols represent 2600 and 2700 K data points, respectively. 2σ error bars are shown. The data points are offset slightly from their true X-values to avoid overlapping of symbols and error bars. 2σ error bars are shown.
